# Dammarane Sapogenins Ameliorates Neurocognitive Functional Impairment Induced by Simulated Long-Duration Spaceflight

**DOI:** 10.3389/fphar.2017.00315

**Published:** 2017-05-29

**Authors:** Xiaorui Wu, Dong Li, Junlian Liu, Lihong Diao, Shukuan Ling, Yuheng Li, Jianyi Gao, Quanchun Fan, Weijia Sun, Qi Li, Dingsheng Zhao, Guohui Zhong, Dengchao Cao, Min Liu, Jiaping Wang, Shuang Zhao, Yu Liu, Guie Bai, Hongzhi Shi, Zi Xu, Jing Wang, Chunmei Xue, Xiaoyan Jin, Xinxin Yuan, Hongxing Li, Caizhi Liu, Huiyuan Sun, Jianwei Li, Yongzhi Li, Yingxian Li

**Affiliations:** ^1^The Key Laboratory of Aerospace Medicine, Ministry of Education, The Fourth Military Medical UniversityXi’an, China; ^2^State Key Laboratory of Space Medicine Fundamentals and Applications, China Astronaut Research and Training CenterBeijing, China; ^3^State Key Laboratory of Proteomics, Beijing Institute of Radiation Medicine, Beijing Proteome Research CenterBeijing, China; ^4^Department of Pharmacy, Beijing Shijitan Hospital, Capital Medical UniversityBeijing, China; ^5^Xiyuan Hospital, China Academy of Chinese Medical SciencesBeijing, China

**Keywords:** dammarane sapogenins, cognitive, space flight, rat models, neurotransmitter, MAPK, PI3K/AKT, BDNF

## Abstract

Increasing evidence indicates the occurrence of cognitive impairment in astronauts under spaceflight compound conditions, but the underlying mechanisms and countermeasures need to be explored. In this study, we found that learning and memory abilities were significantly reduced in rats under a simulated long-duration spaceflight environment (SLSE), which includes microgravity, isolation confinement, noises, and altered circadian rhythms. Dammarane sapogenins (DS), alkaline hydrolyzed products of ginsenosides, can enhance cognition function by regulating brain neurotransmitter levels and inhibiting SLSE-induced neuronal injury. Bioinformatics combined with experimental verification identified that the PI3K-Akt-mTOR pathway was inhibited and the MAPK pathway was activated during SLSE-induced cognition dysfunction, whereas DS substantially ameliorated the changes in brain. These findings defined the characteristics of SLSE-induced cognitive decline and the mechanisms by which DS improves it. The results provide an effective candidate for improving cognitive function in spaceflight missions.

## Introduction

In long-duration spaceflight, astronauts face a special and complex extreme environment that is characterized by microgravity, noise, circadian disturbance, isolation, confinement, and radiation. The spaceflight environment is a challenge to both the physical and the psychological health of humans. Cognition is one of the most important aspects being affected because it influences on efficiency, performance, and even safety during space missions (Fowler et al., 2000; Torre et al., 2012).

In some previous studies, some evidence of cognitive decline was found (Eddy et al., 1998; Shepanek, 2005), but it was still difficult to use the available evidence from actual spaceflight to estimate the true effects, mainly because of the small numbers of subjects (Strangman et al., 2014). Moreover, the ground-based models used in researches simulated mostly only one or two of the environmental factors, such as microgravity (Seaton et al., 2009), isolation and confinement (Basner et al., 2014), or noise (Cheng et al., 2011), which differ greatly from the real spaceflight environment. To the best of our knowledge, only a few attempts have been made to study the influence of a simulated complex spaceflight environment on cognitive function (Ma et al., 2013; Shtemberg et al., 2014), and no comprehensive studies involving complex factors have yet been performed. Simulating spaceflight environment including complex factors are important for systematic study of the effects on cognition.

Different tools and methods have been developed to assess and counteract cognitive deficits, but the effects remain limited and unclear. There is a great need for more safe and effective precautions and countermeasures. Therefore, we are focusing our research on countermeasures and the application of Chinese traditional medicine to help flight crews mitigate the cumulative effects of cognitive impairment and enhance operational performance.

As a tonic, *Panax ginseng* has been widely used in Oriental medicine for several thousands of years and has gained popularity in the West over the last decades. Extensive literature has reported on the bioactivities of ginseng, which contains ginsenosides as its main components, including anti-aging, anti-diabetic, immunoregulatory, anti-cancer, neuroregulatory, wound and ulcer healing activity, etc. (Ru et al., 2015). Ginsenosides are representative pharmaceutical compounds derived from ginseng. Previous studies demonstrated that PPT and PPD are the main hydrolysis metabolites of ginsenosides in the gastrointestinal tract. They can be absorbed more easily and exhibit more potent biological activities (Tawab et al., 2003; Hasegawa, 2004).

**FIGURE 1:**
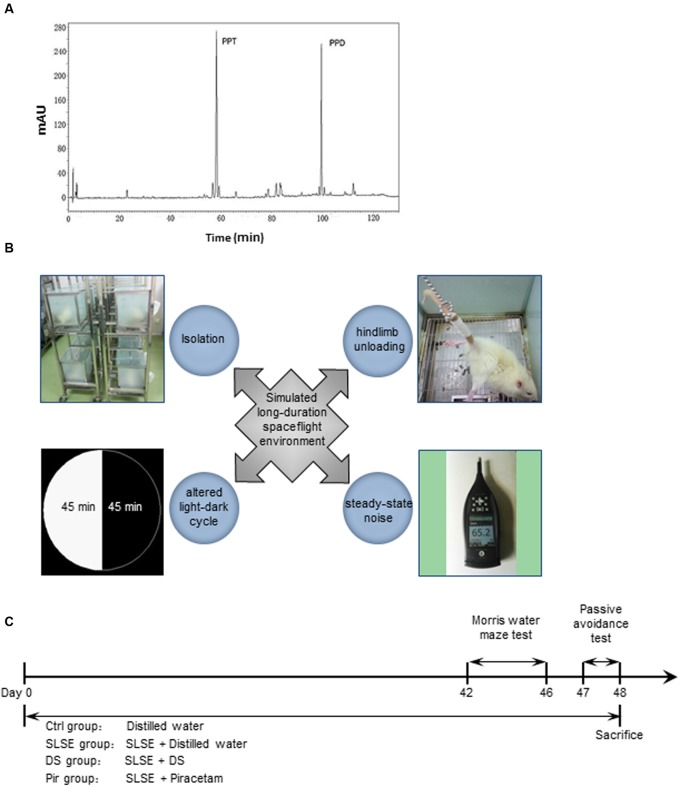
Typical HPLC chromatogram of DS, the four factors of a SLSE, and the experimental schedule. **(A)** Typical HPLC chromatogram of DS. PPT, 20(s)-protopanaxatriol; PPD, 20(s)-protopanaxadiol. **(B)** The four factors included in the SLSE. **(C)** Scheme of the experimental procedure. All of the animals except those in the Ctrl group were exposed to an SLSE until they were sacrificed after behavioral tests. Animals received intragastric administration of distilled water, DS, or piracetam until day 48, when they were sacrificed (*n* = 8 for each group). DS, dammarane sapogenins; SLSE, simulated long-duration spaceflight environment.

Dammarane sapogenins (Patent No. US 6,888014B2, Pegasus Biopark (Dalian) Co. Ltd., Dalian, China), which is mainly composed of PPT and PPD (**Figure 1A**), is an alkaline hydrolysis product of the total ginsenosides extracts derived from the stem and leaf of *P. ginseng*. Previous studies showed that DS has significant activities toward decreasing chemotherapy-induced myelosuppression (Yang et al., 2011) and antidepressant-like effects on chronically restrained rats (He et al., 2013). However, it remains unknown whether DS exerts cognition-enhancing effects similar to those of ginseng, especially in long-duration spaceflight.

In the present study, we used a model of SLSE in rats to investigate the changes in learning and memory. We observed the effects of DS on the SLSE-exposed rats through behavioral experiments, measurement of brain neurotransmitters levels, and brain histopathology analysis. The underlying mechanisms were explored through antibody-based protein microarrays analysis and experimental confirmation. The data indicate that DS attenuated learning and memory deficits in SLSE-exposed rats, which is related to its roles in regulating neurotransmitter levels and preventing neuronal injury. The PI3K-Akt-mTOR and MAPK signaling pathways play an important role in this process. These findings defined the characteristics of SLSE-induced cognitive decline and the mechanisms by which DS improves cognition. DS is an effective candidate for improving cognitive function in spaceflight missions. To the best of our knowledge, this is the first report about the effect of simulating multiple aspects on cognition and about the drug for preventing and treating cognitive impairment in spaceflight missions.

## Materials and Methods

### Animals and Treatments

SPF male Sprague–Dawley rats (180–210 g) were purchased from the Experiment Animal Center of the Academy of Military Medical Sciences (Beijing, China) and bred and maintained at the SPF Animal Research Building of China Astronaut Research and Training Center. The temperature was controlled at 23°C and the animals had free access to food and water. After 7 days of acclimatization in conditions of 12-h light and 12-h dark cycles, the rats were randomly divided into the following four groups (eight animals per group): control (Ctrl), SLSE, SLSE plus DS (DS), and SLSE plus piracetam (Pir) groups.

The rats in the Ctrl group were kept five per cage with a natural light-dark cycle (12-h light and 12-h dark). Other rats were given stimulated SLSE. The rats in DS groups received intragastric administration with DS (50 mg⋅kg^-1^; Pegasus Pharmaceuticals Group Inc, Richmond, BC, Canada) once a day. Those in Pir groups were given Pir (216 mg⋅kg^-1^; Huazhong Pharmaceutical Co. Ltd., Xiangyang, China) by gavage once a day. The Ctrl and SLSE groups were given an equal volume of distilled water, which was the vehicle used for DS and Pir. Behavioral tests were performed from 9:00 to 12:00 after 6 weeks of drug administration. The drug and SLSE administration lasted until the day when behavioral tests finished (**Figure 1C**).

This study was carried out in accordance with the recommendations of guidelines for the use and care of live animals, which was approved by the Animal Care and Use Committee of China Astronaut Research and Training Center.

### SLSE

Simulated long-duration spaceflight environment was established as the method in our previous study (Liu et al., 2015). Four factors were chosen to simulate the spaceflight environment: hind-limb unloading, isolation rearing, steady-state noise, and altered light-dark cycle (**Figure 1B**). Rats were reared individually in cages with frosted glass to simulate isolation and confinement in manned space explorations. The hind-limb-unloading procedure was achieved by tail suspension, as described previously (Morey-Holton and Globus, 2002; Wang et al., 2013), to simulate microgravity in space. Briefly, the rats were individually caged and suspended by the tail using a strip of adhesive surgical tape attached to a chain hanging from a pulley. The rats were suspended at a 30° angle to the floor with only the forelimbs touching the floor, which allowed them to move and access food and water freely. At the same time, 90 min cycles with 45-min light and 45-min dark were generated using an automatic light controller to simulate the light-dark cycle in low-earth-orbit flight. A continuous steady-state noise of 65 ± 5 dB sound pressure level was generated with a white noise generator (UZ-3; Great Wall Electronic Equipment Co., Beijing, China) and passed to a loudspeaker (D40, SAST, Shenzhen, China) by a balancer (DEQ2496; Behringer, Zhongshan, China) and a power amplifier (MP3-50; TAMO, Shenzhen, China). This was to simulate the noise in the spaceflight environment, which is closed to the noise intensity in a manned aircraft (Si et al., 2016). The noise was measured using sound level meters (Type 2270; B&K, Naerum, Denmark) and was applied consistently to rats 12 h a day.

### Behavioral Assessments

Learning and memory were assessed using MWM test and SDPAT. MWM tests were used to evaluate spatial learning and memory (D’Hooge and De Deyn, 2001) with slight modifications from the method reported previously (Shetty et al., 2013). A circular pool (120 cm in diameter, 50 cm in depth) filled with water (30 cm in depth, made opaque by the addition of black ink and maintained at 23 ± 1°C) was divided into four equally spaced quadrants (I, II, III, and IV) with numerous extra maze cues on its walls. A platform (10 cm in diameter) was placed in the center of quadrant III, with its surface 1 cm below the water level. The water maze was placed in a quiet room with dimmed lights. The rats were placed individually in water in one of the three quadrants not containing the platform. They were placed facing the wall around the rim of the pool. The rats that failed to find and stand on the platform within 60 s were guided to the platform and allowed to stay there for 10 s. The place navigation trials were carried out for 4 days (three times a day). On the 5th day, the spatial probe trial was conducted in the absence of the platform. The rats were placed in the water from quadrant I. Their activities were videotaped and analyzed with a custom-built MWM automatic display and analysis processing system, and then the escape latency (times to find the platform) in place navigation trials and the percentage of time spent in the platform quadrant in the spatial probe trial were calculated.

Step-down passive avoidance test was used to measure memory retention and the ability for non-spatial learning and memory with some modifications from the method reported previously (Zarrindast et al., 2006; Bortolatto et al., 2013). The apparatus was a box (30 cm × 30 cm × 40 cm high) with parallel stainless steel bars (0.3 cm diameter spaced 1 cm apart) that was placed on the floor. A cylindrical platform (10 cm in diameter and height) was placed on the center of the grid floor. On the 1st day of the training session, each rat was placed in the box to acclimatize for 3 min, and the bars were then electrified (3 mA, 65 V) for 5 min. Memory retention tests were carried out 24 h later after the training session. Without acclimatization, each rat was placed in the box again with the bars electrified (3 mA, 65 V) for 5 min. The activities were videotaped and analyzed using an SDPAT automatic display and analysis processing system, and then the latency time (time to step onto the platform from the electrified bars), the duration on the platform (over a 4-min period), and error times (the number of times stepped down from the platform during 4 min) were calculated.

### Tissue Processing

1 day after the behavioral tests, rats were deeply anesthetized with chloral hydrate (500 mg⋅kg^-1^) and decapitated to collect brain tissue. Four brains in each group were fixed in 4% paraformaldehyde for histopathological evaluation. The other four brains in each group were dissected into hippocampus and cortices and then were stored at -80°C until analysis. The latter were ground in liquid nitrogen for liquid chromatography-tandem mass spectrometry (LC–MS/MS) analysis, microarray analysis, and western blotting analysis.

### LC-MS/MS Analysis

Liquid chromatography-tandem mass spectrometry method was used to measure seven major neurotransmitters simultaneously in rat brain, including 5-HT, DA, NE, E, Glu, ACh and GABA. The analytical method was carried out as the previous study with some modifications (Huang et al., 2014). Briefly, 20 mg of hippocampus and cortices were homogenized with 9 times the amount of ice-cold normal saline. The homogenate and 3 times the ice-cold acetonitrile was vortex-mixed and placed for 30 min at 4°C, and then centrifuged at 12,000 r⋅min^-1^ at 4°C. The supernatant was collected. 200 μL of the supernatant was mixed with 20 μL internal standard solution (0.3 mg⋅mL^-1^ dihydroxy-benzoic acid), then the mixture (50 μL) was injected into LC–MS/MS system for assay.

Liquid chromatography-tandem mass spectrometry method was run on a Q-Trap 5500 hybrid triple quadrupole-linear ion trap mass spectrometer (SCIEX, Vaughan, ON, Canada) equipped with an electrospray ionization ion source and an Agilent 1290 HPLC system with auto-sampler (Agilent Technologies, Santa Clara, CA, United States). The chromatographic separation was carried out using a TSKgel Amide-80 HPLC column (2 mm × 15 cm, 3 μm, Tosoh, Tokyo, Japan) at 35°C. The mobile phase system was a mixture of acetonitrile-ammonium formate solution (15mmol⋅L^-1^, pH = 5.5, 40:60, v/v) with flow rate of 0.4 mL⋅min^-1^. The injection volume was 5 μL. The instrument was operated in positive electrospray ionization multiple reaction monitoring mode with the following parameter settings: ion spray voltage, 5500 V; gas temperature, 500°C; gas 1, 50 Psi; gas 2, 70 Psi. The multiple reaction monitoring parameters used for analysis were summarized in **Table 1**.

**Table 1:** Parameters of neurotransmitter for MS condition.

Analyte	*Q1* (m/z)	*Q3* (m/z)	Declustering potential (V)	Entrance potential (V)	Collision energy (eV)	Collision cell exit potential (V)
5-HT	177	160	65	12	10	8
DA	154.2	136.6	30	12	15	40
NE	170	152	95	12	15	10
E	184	165.8	40	7	12	15
Glu	148	84	95	4	17	9
GABA	104.1	87	50	10	17	8
ACh	146.1	87.4	160	10	20	30


### Histopathology Analysis

After post-fixing in 4% paraformaldehyde for 48 h, the whole brains were dehydrated and embedded in paraffin. Brain sections (5 μm) were then prepared and subjected to Nissl’s staining. The populations of intact neurons in different sub-regions were counted by a blinded observer.

### Antibody-Based Protein Microarray Analysis

Each group consisted of six samples (including three hippocampi and three cerebral cortices). The KAM-850 antibody microarray (Kinexus, Vancouver, Canada) was used to perform an unbiased characterization of the signaling proteins that were regulated in the different groups, as described previously (Kupcova Skalnikova et al., 2013), in accordance with the manufacturer’s instructions. Briefly, 50 μg of protein lysate from each sample was covalently labeled with a proprietary fluorescent dye. Free dye molecules were then removed by gel filtration. After blocking non-specific binding sites on the array, an incubation chamber was mounted onto the microarray to permit the loading of three samples side by side on the same chip and prevent sample mixing. Following sample incubation, unbound proteins were washed away. The arrays were then scanned using a Scan Array Reader laser array scanner (Perkin-Elmer, Waltham, MA, United States) with a resolution of 10 μm, and the acquired images were quantified using ImaGene 9.0 (BioDiscovery, El Segundo, CA, United States) with predetermined settings for spot segmentation and background correction. *Z* scores were calculated by subtracting the overall mean intensity of all spots within a sample from the raw intensity for each spot and then dividing it by the SD of all of the measured intensities within each sample. *Z* ratios were further calculated by taking the difference between the means of the observed protein *Z* scores and dividing by the SD of all of the differences for that particular comparison. A *Z* ratio above 1.5 was inferred as significant. By converting the corresponding genes to proteins, KEGG enrichment analysis was then performed to detect altered signaling pathways in different groups, as described previously (Kanehisa and Goto, 2000).

The rat protein interaction datasets were downloaded from STRING database v10^1^, and only the high confidence interactions with experimental evidence were selected (Szklarczyk et al., 2015). The network graphs were produced using Cytoscape software (Shannon et al., 2003). Their topological parameters, such as degree centrality, betweenness centrality, clustering coefficients, shortest path lengths, and closeness, were calculated using Cytoscape plug-in Network Analyzer (Assenov et al., 2008). The proteins were then ranked by protein degree.

### Western Blotting

Hippocampal and cortical tissues were extracted using Tissue Protein Extraction Reagent (Thermo, Rockford, IL, United States) plus Protease Inhibitor Cocktail (Roche, South San Francisco, CA, United States) and Phosphatase Inhibitor Mixture (Applygen, Beijing, China) on ice for 30 min. Protein fractions were then collected by centrifugation at 15,000 *g* at 4°C for 10 min, subjected to SDS-PAGE, and transferred to polyvinylidene difluoride membranes. The membranes were blocked with 5% BSA and incubated with specific antibodies overnight. After washing and incubating with horseradish peroxidase-labeled secondary antibodies, the specific bands were visualized using Immobilon Western Chemiluminescent HRP Substrate (Millipore, Billerica, MA, United States). The following antibodies were used: PI3K (rabbit, 1:1000; CST, 4249), p-PI3K (rabbit, 1:1000; CST, 2971), AKT (rabbit, 1:1000; CST, 9272), p-Akt (rabbit, 1:1000; CST, 9271), mTOR (rabbit, 1:1000; CST, 2983), p-mTOR (rabbit, 1:1000; CST, 2971), CREB (rabbit, 1:1000; CST, 9197), p-CREB (rabbit, 1:1000; CST, 9198), BDNF (sheep, 1:1000; Abcam, ab75040), JNK (mouse, 1:1000; CST, 3708), p-JNK (mouse, 1:1000; CST, 4668), p38 (rabbit, 1:1000; CST, 8690), p-p38 (rabbit, 1:1000; CST, 4511), and GAPDH (mouse, 1:1000; Santa Cruz, sc-47724). The ratio of the protein band intensities relative to that of GAPDH was calculated for each sample using Image J software (US National Institutes of Health, Bethesda, MD, United States).

### Statistical Analysis

All data are presented as means ± SEM and were analyzed using GraphPad Prism 5.0 software (GraphPad Software, San Diego, CA, United States). Multiple comparisons were performed using one-way ANOVA followed by Bonferroni’s *post hoc* test. *P* values less than 0.05 were considered statistically significant.

## Results

### DS Enhanced Cognition in Rats Exposed to SLSE in Behavioral Experiments

**FIGURE 2:**
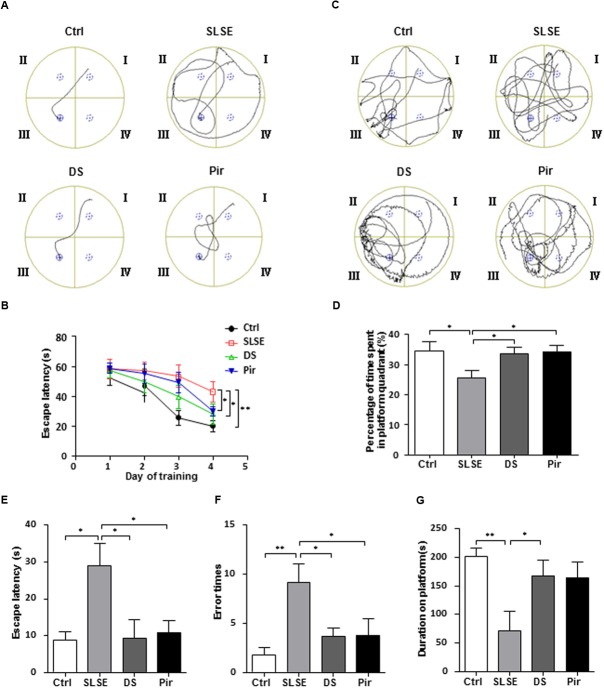
Simulated long-duration spaceflight environment induced defects in learning and memory behaviors and DS reversed these effects. The efficiency of spatial learning and memory in the MWM test is shown in **(A–D)**. **(A)** Representative searching swimming paths on the 4th day of place navigation trials. **(B)** Escape latency to find the platform during the 4 days of place navigation trials. **(C)** The representative searching swimming paths in the spatial probe trial performed on the 5th day of the MWM trial. **(D)** The percent time spent in the platform quadrant in the spatial probe trial on the 5th day of the MWM trial. The efficiency of passive avoidance in SDPAT is shown in **(E–G)**. **(E)** The escape latency to step onto the elevated platform from electrified bars on the 2nd day of SDPAT. **(F)** Error times that the rats stepped down from the platform during 4 min on the 2nd day of SDPAT. **(G)** Total duration on platform during 4 min on the 2nd day of SDPAT. Data are expressed as means ± SEM. All of the differences were analyzed using one-way ANOVA with Bonferroni’s *post hoc* test (*n* = 8 for each group, ^∗^*p* < 0.05, ^∗∗^*p* < 0.01). MWM, Morris water maze; SDPAT, step-down passive avoidance test; Pir, piracetam.

The common behavioral methods MWM tests and SDPAT were used to assess the cognitive effects of SLSE exposure in rats (**Figure 2**). In the MWM test, representative searching swimming paths on the 4th day of place navigation trials displayed clear differences among groups (**Figure 2A**). Escape latencies to the hidden platform from quadrant I decreased in all groups over 4 days of the place navigation test (**Figure 2B**). SLSE-treated rats had higher latencies than the control, and DS-and piracetam-treated rats had lower latencies than the rats in the SLSE group on the 4th day. **Figure 2C** shows that SLSE-treated rats had a tendency to swim everywhere in the MWM, whereas rats in other groups preferred to move in the platform quadrant in the spatial probe test performed on the 5th day. The percentage of time spent in the platform quadrant was decreased in SLSE-treated rats compared with that in the control, and was increased in the DS and Pir groups compared with that in the SLSE group (**Figure 2D**). These results suggest that SLSE can lead to memory loss and cognitive decline, and that DS and piracetam can improve spatial learning and memory.

In SDPAT, SLSE significantly increased the latency time and error times compared with those in the control group, and DS and piracetam decreased the latency time and error times compared with those in the SLSE group (**Figures 2E,F**). Meanwhile the total duration spent on the platform was decreased in the SLSE group compared with that in the control, and was increased in the DS group compared with that in the SLSE group; however, there was no significant difference between the SLSE and Pir groups (**Figure 2G**). These results indicate that SLSE can decrease the efficiency of passive avoidance, and that the cognition-enhancing effects of DS are similar to that of piracetam. Above all, these findings suggest that SLSE can induce neurocognitive functional impairment in rats, and that DS has cognition-enhancing effects in SLSE-exposed rats.

### DS Altered the Neurotransmitter Content in the Hippocampus and Cortices of Rats Exposed to SLSE

**FIGURE 3:**
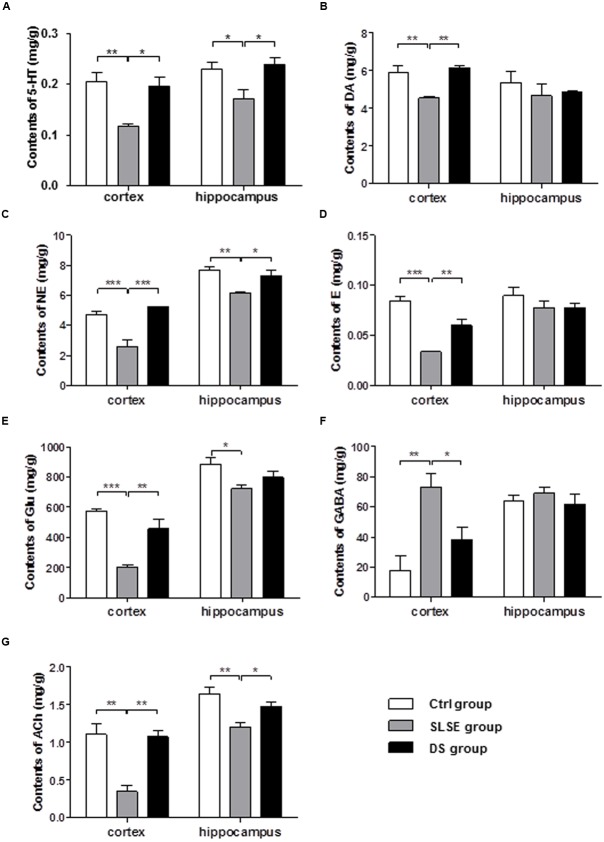
Dammarane sapogenins alters the neurotransmitter content in the cortex and hippocampus of rats exposed to SLSE. Seven neurotransmitters were measured simultaneously using LC-MS/MS. The effects of DS on the contents of four monoamine neurotransmitters were shown: 5-HT **(A)**, DA **(B)**, NE **(C)**, and E **(D)**. The contents of two amino acid neurotransmitters, Glu **(E)** and GABA **(F)**, were measured at the same time. The content of the cholinergic neurotransmitter ACh is shown in **(G)**. Data are expressed as means ± SEM. All of the differences were analyzed using one-way ANOVA with Bonferroni’s *post hoc* test (*n* = 4 for each group, ^∗^*p* < 0.05, ^∗∗^*p* < 0.01, ^∗∗∗^*p* < 0.001).

Neurotransmitters are closely linked to cognitive function. A rapid and sensitive LC–MS/MS method was used to measure seven major neurotransmitters simultaneously in the rat brain (**Figure 3**), including four monoamine (**Figures 3A–D**), two amino acid (**Figures 3E,F**), and ACh (**Figure 3G**). In the cerebral cortices, compared with those in the control group, the contents of 5-HT, DA, NE, E, Glu, and ACh (**Figures 3A–E,G**) were decreased in the SLSE group, whereas the GABA content (**Figure 3F**) was increased. The GABA content (**Figure 3F**) in the cerebral cortices was decreased in the DS treatment group compared to that in the SLSE group, while the levels of all other markers were increased. In the hippocampus, the changes in the 5-HT, NE, and ACh contents were the same as those in the cerebral cortices (**Figures 3A,C,G**); The other neurotransmitters had no significant changes (**Figures 3B,D–F**). These results show that DS had significant effects on neurotransmitter levels in the SLSE-exposed rat brain.

### DS Affected the Numbers and Arrangement of Neurons in the Hippocampus and Cortices of SLSE-Exposed Rats

**FIGURE 4:**
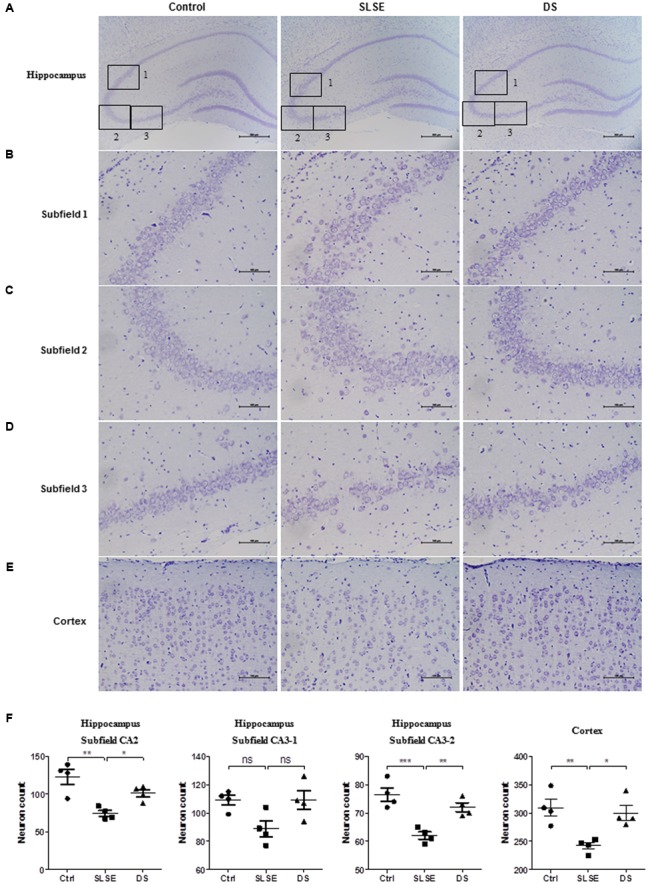
Dammarane sapogenins affected the numbers and arrangements of neurons in the hippocampus and cortex of SLSE-exposed rats. Nissl’s staining results **(A–E)** and the corresponding analyses **(F)** are shown (*n* = 4 for each group). Subfields 1, 2, and 3 (**B–D**, scale bar, 100 μm) are subregions of the whole hippocampus (**A**, scale bar, 500 μm). The effects of DS on the cortex are shown in **(E)** (scale bar, 100 μm). **(F)** The numbers of intact neurons in the different sub-regions were counted by an investigator who was blinded to the sample identity. Data are expressed as means ± SEM. The differences were analyzed using one-way ANOVA with Bonferroni’s *post hoc* test (^∗^*p* < 0.05, ^∗∗^*p* < 0.01, ^∗∗∗^*p* < 0.001).

To assess the effects of SLSE and DS on neurons, Nissl’s staining and microphotography were used to study the changes in the hippocampal and cortical tissues in the different groups (**Figure 4**). Compared with those in the control and DS groups, neurons in the hippocampal subfields 1, 2 and 3 (**Figures 4A–D**) and cerebral cortices (**Figure 4E**) of SLSE-exposed rats were larger in the intercellular space, loosely and irregularly arranged, and weakly stained, indicating that they were injured (**Figures 4A–E**). There were significantly fewer neurons in the hippocampal subfields 1, 3 and cerebral cortices in SLSE-exposed rats than in the control and DS groups (**Figure 4F**). According to the hippocampal regionalism, subfields 1, 2 and 3 are in the region of CA3. DS significantly increased the number of neurons in the hippocampus CA3 and cortex and prevented neuronal injury in SLSE rats.

### DS Affected Protein Expression in the Brains of Rats Exposed to SLSE

Protein antibody microarrays are the most efficient and economical way to analyze multiple samples or proteins at the same time (Wingren and Borrebaeck, 2004). To investigate further the underlying molecular mechanism of action of DS in SLSE rats, Kinex^TM^ antibody microarrays were used to profile protein expression in rat brains. A total of 854 molecules were detected in the microarray.

**FIGURE 5:**
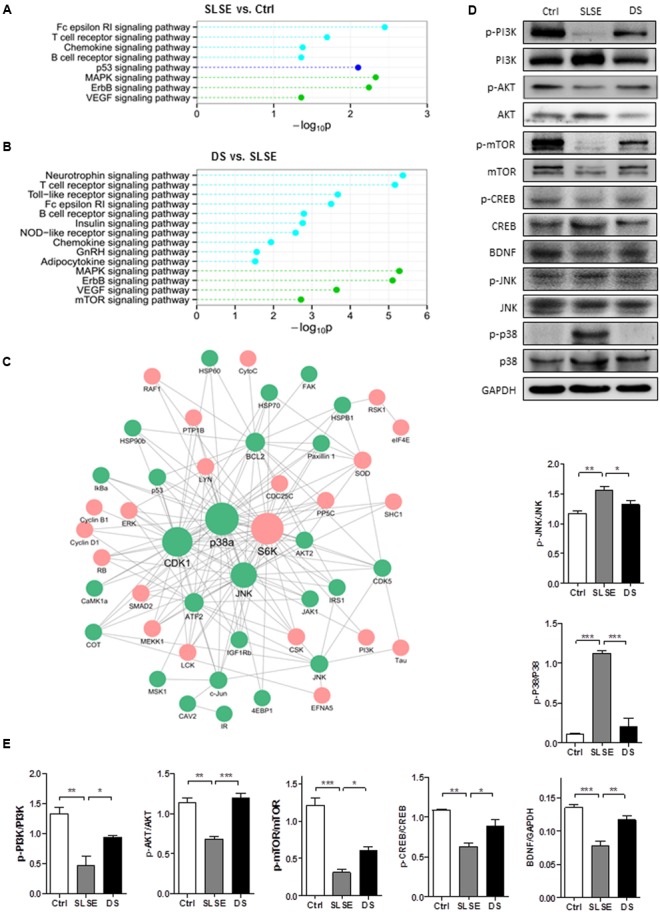
Dammarane sapogenins affected the protein expression in the hippocampus of SLSE-exposed rats. KinexTM antibody microarrays were used to profile protein expression in the brains of SLSE, DS-treated, and control rats (*n* = 3 for each group). The altered signaling pathways were identified using KEGG enrichment analysis. Statistically significant value “*p*” was transformed to –log10P. The larger the “–log10P,” the more significant the difference is. Pathways within the same category are marked in the same color. Signaling pathways that differed between the Ctrl and SLSE groups in the hippocampus are shown in **(A)**, and the signaling pathways that differed between the DS and SLSE groups are shown in **(B)**. There were 51 differential expressed proteins whose trends in the Ctrl and DS groups compared with the SLSE group were consistent. By identifying the interologs, a hippocampus protein interaction network **(C)** was constructed with 165 interactions among 51 differentially expressed proteins. The proteins that were up-regulated by DS are marked with red circles, and those that were down-regulated by DS are marked with green circles. The proteins with more interactions with others were represented with larger circles. **(D)** Hippocampal tissue lysates were analyzed by western blotting with the indicated antibodies. **(E)** The corresponding statistical graphs of western blotting analyses. Data are expressed as means ± SEM. The differences were analyzed using one-way ANOVA with Bonferroni’s *post hoc* test (^∗^*p* < 0.05, ^∗∗^*p* < 0.01, ^∗∗∗^*p* < 0.001).

**FIGURE 6:**
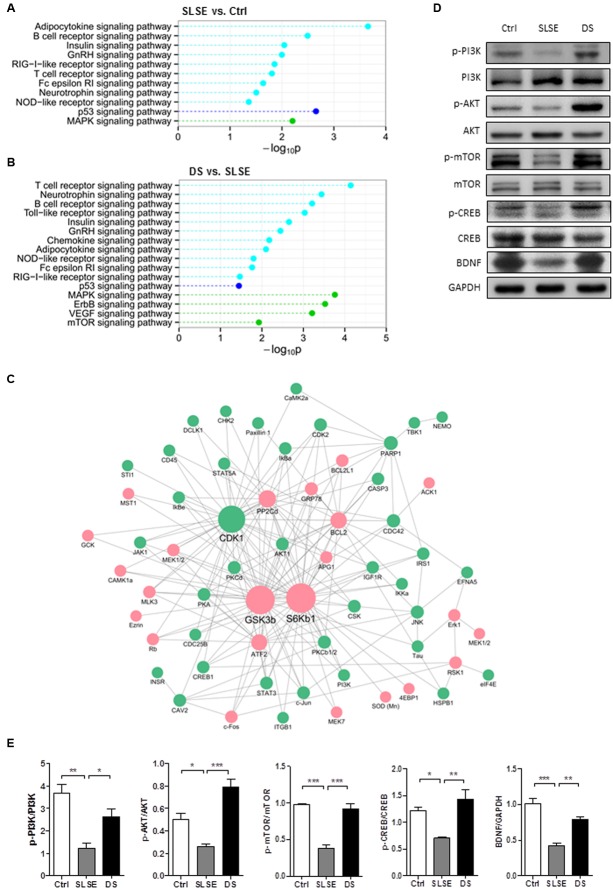
Dammarane sapogenins affected the protein expression in the cortices of SLSE-exposed rats. KinexTM antibody microarrays were used and analyzed as described for **Figure 6** (*n* = 3 for each group). The signaling pathways altered between the Ctrl and SLSE groups in cortices are shown in **(A)**. Those altered between the DS and SLSE groups are shown in **(B)**. There were 65 differentially expressed proteins whose trends in the Ctrl and DS groups compared with the SLSE group were consistent. By identifying the interologs, a cortex protein interaction network **(C)** was constructed with 193 interactions among 65 differentially expressed proteins. The proteins that were upregulated by DS are marked with red circles, and those that were downregulated by DS are marked with green circles. The proteins with more interactions with others were represented with larger circles. **(D)** Cortical tissue lysates were analyzed by western blotting with the indicated antibodies. **(E)** The corresponding statistical graphs of western blotting analyses. Data are expressed as means ± SEM. The differences were analyzed using one-way ANOVA with Bonferroni’s *post hoc* test (^∗^*p* < 0.05, ^∗∗^*p* < 0.01, ^∗∗∗^*p* < 0.001).

In the hippocampus, KEGG pathway enrichment analysis showed that 8 pathways were significantly altered in the SLSE group compared with those in the control (**Figure 5A**) and 14 pathways were significantly altered in the DS group compared with those in the SLSE group (**Figure 5B**). In the cerebral cortices, the numbers of pathways altered in the respective groups were 11 (**Figure 6A**) and 16 (**Figure 6B**). The MAPK and neurotrophin signaling pathways were significantly altered in most groups; their “–log_10_P” values were larger than those of most other pathways, which suggested that they could be most strongly related to the changes in nervous function.

There were 51 differentially expressed proteins whose trends of change in the Ctrl and DS groups compared with the SLSE group were consistent. A hippocampus protein interaction network was constructed with 165 interactions among the 51 differentially expressed proteins based on STRING database (**Figure 5C**). In this network, there were more proteins connecting with p38a, CDK1, JNK, and S6K than with the others; therefore, these may be potential target proteins of DS. Similarly, a cortex protein interaction network composed of 193 interactions among 65 differentially expressed proteins was constructed (**Figure 6C**). The network revealed that CDK1, GSK3b, S6Kb1, Bcl-2, and ATF2 proteins were more likely than the others to play important roles in the cognition-enhancing effects of DS.

To validate further which pathways are influenced by DS in the cerebral cortex and hippocampus of SLSE rats, the main proteins in the MAPK and neurotrophin signaling pathways and some other key proteins in the protein interaction networks were analyzed using western blotting (**Figures 5D,E**, **6D,E**). As shown in **Figures 5D**, **6D**, both in the hippocampus and in the cortices, the expression of p-PI3K, p-AKT, and p-mTOR was significantly decreased in the SLSE group compared with that in the control group, and increased in the DS group compared with that in the SLSE group. In the two tissues, the expression of p-CREB and BDNF was significantly decreased in the SLSE group compared with that in the control, and increased in the DS group compared with that in the SLSE group. But only in the hippocampus, the expression of p-JNK and p-p38 was increased in the SLSE group compared with that in the control group, and decreased in the DS group compared with that in the SLSE group. There were no significant changes in the expression of p-JNK and p-p38 in the cortices (Supplementary Figure 1).

## Discussion

In this study, we examined the changes in learning and memory in rats after 42 days of SLSE exposure, the countermeasure effects of DS, and its underlying molecular mechanism of action. The results demonstrated that SLSE significantly impaired learning and memory and that DS exhibited preventive and therapeutic effects on SLSE-induced neurocognitive functional impairment.

For the first time, four factors of the compound spaceflight environment were comprehensively simulated to study the cognition in spaceflight: microgravity, isolation, noise, and circadian rhythm desynchrony. Behavioral procedures were then used to assess the effects of SLSE exposure on cognition in rats and also evaluate the protective effects of DS. Piracetam, a nootropic drug for CNS disorders that is effective at improving memory, was used as the control drug (Malykh and Sadaie, 2010).

The MWM has proven to be a robust and reliable test to evaluate spatial learning and memory for rodents. Rodents relied on distal cues to navigate from start locations around the perimeter of an open circular pool to locate a submerged escape platform. Spatial learning is assessed across repeated trials and reference memory is determined by preference for the platform area when the platform is absent (Vorhees and Williams, 2014). In this study, the increased escape latency and decreased percentage of time in the platform quadrant in the MWM test indicated that SLSE exposure induced detrimental neurocognitive deficits in spatial learning and memory, consistent with the findings in previous reports (Ma et al., 2013). As a method for evaluating passive avoidance- and escape-learning responses simultaneously, the SDPAT was developed for the study of learning and memory (Kameyama et al., 1986; Bortolatto et al., 2013). The escape latency in the retention test, the error time of stepping down from the platform, and the duration on the platform are good parameters of learning and memory performance. In this study, SLSE exposure increased the latency time and error times and decreased the duration spent in the platform in SDPAT, suggesting that SLSE exposure reduced memory retention and non-spatial learning and memory. DS-treated rats displayed similar behavior to controls, which suggests that it were resistant to SLSE-induced learning and memory dysfunction. Behavioral results show that DS had significant activities toward improving SLSE-induced cognitive dysfunction in rats, similar to its effects founded in previous studies investigating other cognitive dysfunction models caused by sleep interruption (Kong et al., 2013) and scopolamine (Wu et al., 2015).

In the mammalian brain, multiple learning and memory systems interact with each other in different ways to influence and/or control behavior (McDonald and Hong, 2013). Among them, hippocampus and cortices play the most prominent role in learning and memory. The system in the hippocampus not only plays an important role in the transformation of short-term memory into long-term memory, but also is implicated in episodic memory and spatial memory (Squire and Zola-Morgan, 1991; Rolls, 2000). CA3 is extremely sensitive to stress, long-term chronic stress can lead to neuronal deformation and loss in the region (Sapolsky, 2000). The cerebral cortex is the superior nerve center of learning and memory. The brain systems in the frontal and temporal cortices are involved in short term memory (Rolls, 2000). The prefrontal cortex also performs similar functions for both auditory and visual working memory (Plakke and Romanski, 2016). The primate orbitofrontal cortex is involved in representing rewards and punishers, and in learning stimulus-reinforcer associations. The temporal cortical visual areas are involved in learning invariant representations of objects (Rolls, 2000). Primary auditory cortex has a function in behavioral memory (Weinberger, 2015). The parietal cortex also associated with auditory working memory (Zimmermann et al., 2016). The medial temporal lobe, the prefrontal cortex and sensory association cortices are involved in recognition memory (Brown et al., 2010). The ventral premotor cortex processes both recent sensory information and that from long-term memory to decide and evaluate the behavior of previous decisions. This process may serve for learning and thus adapting future behavior to environmental demands (Acuna et al., 2010). The cerebral cortex is also easy to be injured in chronic stress with decrease of neuron count and size. Nissl bodies are the characteristic structure of neuron, which reflect the function state of cell and is closely related to emotion and cognition (Enna and Bowery, 2004). Because of their prominent roles in learning and memory, we detect the tissues both in the hippocampus and in the cortices.

Neurotransmitters play an important role in cognitive function. They mediate intracellular signaling transduction systems through their receptors, thus regulate almost all nervous system functions including learning and memory. 5-HT, DA, NE, and E are monoamine neurotransmitters that can enhance the excitability of brain and learning and memory ability (Stuchlik et al., 2007; Roozendaal et al., 2008; Perez-Garcia and Meneses, 2009; Seyedabadi et al., 2014). 5-HT is a neurotransmitter that plays an important role in neuronal plasticity, its receptor 5-HT1A induces neurogenesis and synapse formation, by modulation of the neuronal cytoskeleton through MAPK and PI3K/Akt signaling pathways (Rojas and Fiedler, 2016). D1-like DA receptor can activate p38MAPK and JNK by PKA, thus mediate MAPK pathways (Goldman-Rakic et al., 2004), which is associated with learning. Another study found DA induces a PI3K-independent activation of Akt in striatal neurons (Brami-Cherrier et al., 2002). The role of NE and adrenergic neurons is very broad in the brain, almost involving in the regulation of all brain functions, including learning and memory, attention, awareness, alertness, mood, etc. E can activate alpha and beta receptors, and NE activates alpha receptors mostly. As G protein-coupled receptors, their activation can cause the changes of many downstream pathways including PI3K/Akt, JNK, p38MAPK, ERK, BDNF, etc., thus play physiological functions (Zhong and Minneman, 1999; Yanagawa et al., 2010; Day et al., 2014). As main excitatory neurotransmitter that is present at the highest levels in the mammalian brain, Glu plays important roles in learning and memory, synaptic plasticity, and brain development (Collingridge and Singer, 1990). AMPA receptors of Glu can coupled with MAPK, activating MAPK neurotrophin signaling pathways and increase the expression of BDNF (Legutko et al., 2001). GABA is an important inhibitory neurotransmitter in the brain. Excitatory actions of GABA increase BDNF expression via a MAPK-CREB-dependent mechanism in developing neurons (Obrietan et al., 2002; Fukuchi et al., 2014). As a cholinergic neurotransmitter, ACh promotes certain synaptic plastic processes underlying the formation of memories (Rasch et al., 2009) and thereby plays crucial roles in learning and memory (Winkler et al., 1995). GABA is the major inhibitory neurotransmitter that negatively regulates learning and memory function (Farr et al., 2000). As own receptors and heterologous receptors, nicotinic ACh receptor positive regulate the release of Ach, NE, DA, Glu, GABA, etc. Its activation can resulted in significant increase of intracellular Ca^2+^ level, thus downstream MAPK signaling pathways are activated and play roles (Sacco et al., 2004). Here, DS prevented the decrease in NE, E, DA, 5-HT, ACh, and Glu and the increase in GABA in the cerebral cortex. In the hippocampus, only the 5-HT, NE, and ACh contents were affected with the same trends as those observed in the cortex. These results suggest that DS can inhibit the abnormal changes in many neurotransmitters and therefore prevent SLSE-impaired learning and memory. Previous studies revealed that hippocampal NE levels, cortical 5-HT levels, and DA system activity were decreased in space or simulated microgravity (Blanc et al., 1998; Liu et al., 2010; Popova et al., 2015). We identified novel changes in neurotransmitters in simulated spaceflight that need to be further validated.

In spaceflight, the modification of various intrinsic and extrinsic factors can lead to alterations in cerebral morphology and function. Specifically, the number of axodendrite synapses and cortical synapses is significantly reduced, and the cross-sectional synaptic length and the density of the apoptotic cells are significantly larger (DeFelipe et al., 2002; Krasnov and D’Iachkova, 2003; Latchney et al., 2014). In the current study, Nissl’s staining indicated that DS treatment efficiently prevented the neurons of SLSE rats from undergoing injury-induced structural and number changes. These results suggest that DS can protect neurons from injury in SLSE.

To explore further the underlying mechanism behind DS-induced improved learning and memory, a protein microarray was used to screen the altered proteins and their related pathways, and western blotting was used to validate the pathways and proteins. The results demonstrated that PI3K-Akt-mTOR pathways, CREB and BDNF were significantly reduced both in the hippocampus and in the cortices under SLSE conditions. After DS treatment, the levels of the above components were clearly up-regulated. In the hippocampus and cortices, both mTOR and CREB are key regulators in learning and memory (Graber et al., 2013; Kida and Serita, 2014; Giovannini et al., 2015). mTOR is an evolutionarily conserved protein kinase that regulates cell growth, proliferation, and survival by increasing protein translation in response to neuronal activity and thereby modulating synaptic plasticity and long term memory (LTM) formation (Graber et al., 2013). Here, we found that mTOR phosphorylation was clearly reduced by SLSE but was enhanced by DS treatment in both the hippocampus and the cortices. CREB-dependent transcription is essential for many forms of learning and memory (Kida and Serita, 2014). CREB exerts its effects by regulating its targets such as BDNF and c-Fos. The phosphorylation of CREB at Ser133 is an important step in induction of the gene expression that is essential to learning (Leal et al., 2015). Among all neurotrophins, brain-derived neurotrophic factor (BDNF) stands out for its high level of expression in the brain and its potent effects on synapses, especially in hippocampus and cortices. The main function of BDNF in the adult brain is to regulate synapses and improve cognitive function, with structural and functional effects ranging from short-term to long-lasting, on excitatory or inhibitory synapses, in many brain regions (Lu et al., 2014). The current results revealed that DS could increase CREB phosphorylation under SLSE conditions and promote the expression of BDNF in both the hippocampus and the cortices. The study found that DS ameliorated SLSE-induced neurocognitive functional impairment by regulating PI3K-Akt-mTOR pathways, CREB and BDNF in cortices and hippocampus.

But the changes of MAPK pathways occurred only in hippocampus in our study. The results revealed that p38 and JNK were activated by SLSE and their phosphorylation was reduced after DS treatment in hippocampus but not in cortices. JNK and p38 are key players in the regulation of synaptic plasticity and are involved in neuronal apoptosis (Thomas and Huganir, 2004; Hosseini et al., 2010; Ryu and Lee, 2016). The activation of p38 activation by Ras-GRF1 has been implicated in the induction of long-term depression (Giese and Mizuno, 2013). In addition, JNK activation negatively regulates long term memory (LTM) formation and appears to promote memory extinction (Giese and Mizuno, 2013). The study found that DS ameliorated SLSE-induced neurocognitive functional impairment by regulating the levels of p38 and JNK in hippocampal.

**FIGURE 7:**
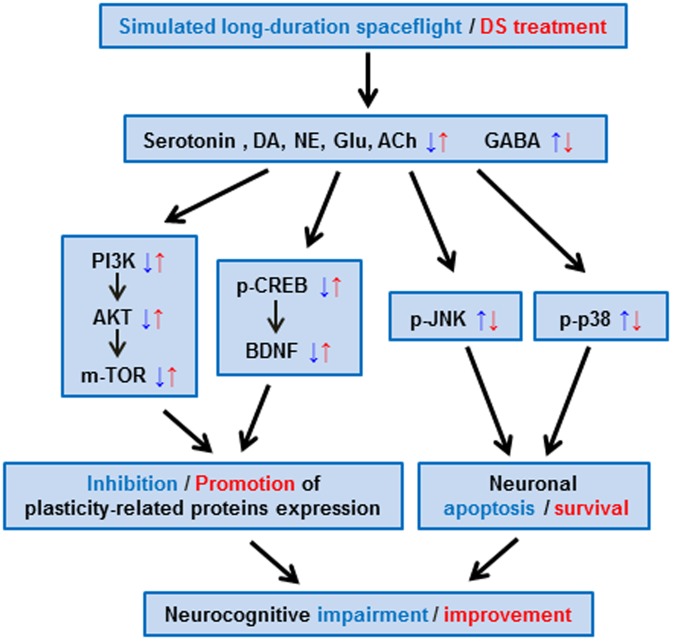
Potential mechanisms of DS in ameliorating the neurocognitive dysfunction induced by simulated long-duration spaceflight in rats. The effects of SLSE were indicated in blue, and those of DS were indicated in red. DS regulated neurotransmitter content changed by simulated long-duration spaceflight. Neurotransmitters mediate intracellular signaling transduction systems through receptors, thus regulate learning and memory. By activating the PI3K-Akt-mTOR pathways and increasing CREB phosphorylation and expression of BDNF, DS promoted the plasticity-related proteins expression. By inhibiting the phosphorylation level of JNK and p38, DS inhibited neuronal apoptosis. DS ameliorate neurocognitive functional impairment induced by simulated long-duration spaceflight.

This study revealed that SLSE reduced learning and memory ability, whereas DS had a protective effect (**Figure 7**). DS regulated neurotransmitter content affected by simulated long-duration spaceflight. Neurotransmitters mediate intracellular signaling transduction systems. In this study, by activating the PI3K-Akt-mTOR pathways and increasing, DS promoted the plasticity-related proteins expression. By inhibiting the phosphorylation level of JNK and p38, DS inhibited neuronal apoptosis. DS ameliorate neurocognitive functional impairment induced by simulated long-duration spaceflight. Although the molecular targets need to be clarified further in the future, DS is a potential countermeasure against long-term spaceflight-induced cognition dysfunction.

## Author Contributions

XW and DL designed and performed the majority of the experiments, analyzed data and prepared the manuscript. JlL analyzed protein microarray data. LD built proteinprotein interaction network graphs. SL and YhL assisted with western blotting analysis. JG and QF helped with intragastric administration. ML, WS, and QL helped with the construction of SLSE model in rats. DZ, GZ, and DC helped with extraction of proteins. JpW, SZ, YuL, GB, HzS, ZX, and JiW helped with the behavioral tests. CX, XJ, XY, HL, and CL helped with brain tissue isolation and blood collection. HyS and JwL helped with electron microscopy observation. YxL and YzL supervised the project. XW and YxL wrote most of the manuscript. All authors have read and approved the final manuscript.

## Conflict of Interest Statement

The authors declare that the research was conducted in the absence of any commercial or financial relationships that could be construed as a potential conflict of interest. The reviewer AJL and handling Editor declared their shared affiliation, and the handling Editor states that the process nevertheless met the standards of a fair and objective review.

## Footnotes

1 http://string-db.org

## Abbreviations

5-HT: 5-hydroxytryptamine
ACh: Acetylcholine
ACTH: adrenocorticotropic hormone
ANS: autonomic nervous system
CAT: catalase
CNS: central nervous system
CORT: corticosterone
DA: dopamine
DS: Dammarane sapogenins
E: epinephrine
GABA: γ-aminobutyric acid
Glu: glutamic acid
HPA: hypothalamic-pituitary-adrenal
HPLC: high-performance liquid chromatography
KEGG: Kyoto Encyclopedia of Genes and Genomes
LC-MS/MS: liquid chromatography-tandem mass spectrometry method
LTM: long time memory
MDA: malondialdehyde
MWM: Morris water maze
NE: norepinephrine
PNS: parasympathetic nervous system
PPD: 20(s)-protopanaxadiol
PPT: 20(s)-protopanaxatriol
SD: standard deviation
SDPAT: step-down passive avoidance test
SLSE: simulated long-duration spaceflight environment
SNS: sympathetic nervous system
SOD: superoxide dismutase
T-AOC: total antioxidant capacity
