# Supplementary figures and images for: Dammarane Sapogenins Ameliorates Neurocognitive Functional Impairment Induced by Simulated Long-Duration Spaceflight
Source: Front Pharmacol. 2017 May 29;8:315. doi: 10.3389/fphar.2017.00315 (PMC5446991; doi:10.3389/fphar.2017.00315)

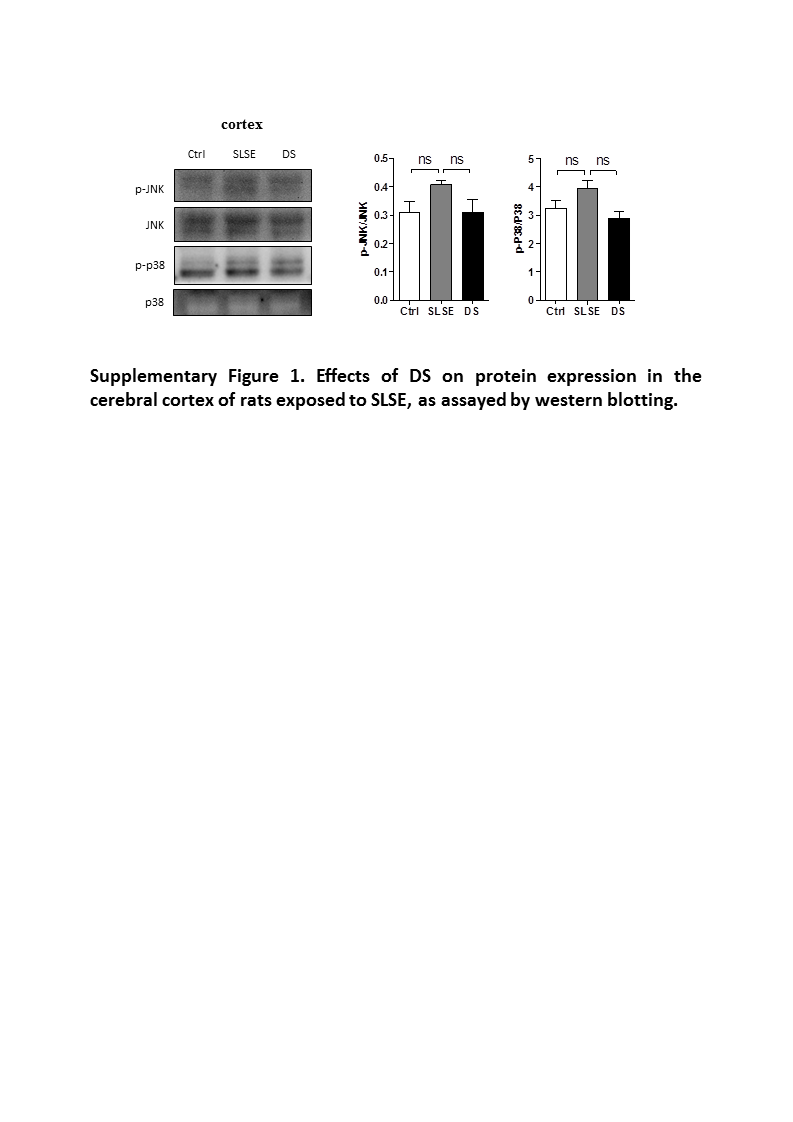

Supplement: Supplementary file 1 [file Image_1.tif]
